# Birth Outcomes after the Fukushima Daiichi Nuclear Power Plant Disaster: A Long-Term Retrospective Study

**DOI:** 10.3390/ijerph14050542

**Published:** 2017-05-19

**Authors:** Claire Leppold, Shuhei Nomura, Toyoaki Sawano, Akihiko Ozaki, Masaharu Tsubokura, Sarah Hill, Yukio Kanazawa, Hiroshi Anbe

**Affiliations:** 1 Global Public Health Unit, School of Social and Political Science, University of Edinburgh, George Square, Edinburgh EH8 9LD, UK; 2 Department of Research, Minamisoma Municipal General Hospital, Minamisoma, Fukushima 975-0033, Japan; S.E.Hill@ed.ac.uk; 3 Department of Global Health Policy, Graduate School of Medicine, The University of Tokyo, 7-3-1, Hongo, Bunkyo-ku, Tokyo 113-0033, Japan; s-nomura@m.u-tokyo.ac.jp; 4 Department of Epidemiology and Biostatistics, School of Public Health, Imperial College London, Norfolk Place, London W2 1PG, UK; 5 Department of Surgery, Minamisoma Municipal General Hospital, Minamisoma, Fukushima 975-0033, Japan; toyoakisawano@gmail.com (T.S.); ozakiakihiko@gmail.com (A.O.); 6 Department of Radiation Protection, Minamisoma Municipal General Hospital, Minamisoma, Fukushima 975-0033, Japan; tsubokura-tky@umin.ac.jp (M.T.); k-yukio@bz04.plala.or.jp (Y.K.); 7 Department of Obstetrics and Gynecology, Minamisoma Municipal General Hospital, Minamisoma, Fukushima 975-0033, Japan; hiroshi-anbe@bz04.plala.or.jp

**Keywords:** disasters, maternal and perinatal health, birthweight, public health, Fukushima

## Abstract

Changes in population birth outcomes, including increases in low birthweight or preterm births, have been documented after natural and manmade disasters. However, information is limited following the 2011 Fukushima Daiichi Nuclear Power Plant Disaster. In this study, we assessed whether there were long-term changes in birth outcomes post-disaster, compared to pre-disaster data, and whether residential area and food purchasing patterns, as proxy measurements of evacuation and radiation-related anxiety, were associated with post-disaster birth outcomes. Maternal and perinatal data were retrospectively collected for all live singleton births at a public hospital, located 23 km from the power plant, from 2008 to 2015. Proportions of low birthweight (<2500 g at birth) and preterm births (<37 weeks gestation at birth) were compared pre- and post-disaster, and regression models were conducted to assess for associations between these outcomes and evacuation and food avoidance. A total of 1101 live singleton births were included. There were no increased proportions of low birthweight or preterm births in any year after the disaster (merged post-disaster risk ratio of low birthweight birth: 0.98, 95% confidence interval (CI): 0.64–1.51; and preterm birth: 0.68, 95% CI: 0.38–1.21). No significant associations between birth outcomes and residential area or food purchasing patterns were identified, after adjustment for covariates. In conclusion, no changes in birth outcomes were found in this institution-based investigation after the Fukushima disaster. Further research is needed on the pathways that may exacerbate or reduce disaster effects on maternal and perinatal health.

## 1. Introduction

Perinatal health is a crucial aspect of public health. Birth outcomes, measureable as birthweight and gestational age at birth, have been found to predict both short- and long-term health trajectories of the neonate; a topic which has gained significant attention in the field of epidemiology [1,2,3,4]. A host of factors can influence birth outcomes, including maternal medical history, environmental and behavioural factors, and sociodemographic factors such as ethnicity, age and marital status [5,6]. Evidence has additionally grown for associations between external stressors and adverse birth outcomes [7], opening new discussions on the broad determinants of health at birth [8].

Disasters are one type of external stressor associated with changes in population birth outcomes. Increases in low birthweight births have been documented after natural disasters, chemical disasters and terrorism, with or without concurrent increases in preterm births [9,10,11,12,13,14,15,16,17,18,19,20]. Post-disaster changes in birth outcomes are thought to be mediated through maternal exposure to environmental toxins or disaster-related psychosocial stress, yet an area that remains unclear is the timeframe between exposure and outcome [9,21]. Most studies to date have focused on women who were pregnant at the time of a disaster, yet there is also evidence for increased prevalence of low birthweight and preterm births lasting for years post-disaster [10,22]. This finding is consistent with growing evidence that stressful life events prior to conception can increase the risk of delivering a low birthweight neonate later in life [23,24], and highlights the possibility that disasters may not only have immediate health impacts, but additionally lead to long-term changes in the birth outcomes of affected populations.

On 11 March 2011, Northeast Japan was struck by an earthquake and tsunami, triggering a nuclear disaster at Fukushima Daiichi Nuclear Power Plant. In contrast to the relatively immediate destruction of the earthquake and tsunami, the nuclear disaster has led to long-term societal changes such as prolonged evacuation [25], and changing health risks have been observed in affected populations [26,27,28]. Issues of stigma, radiation-related anxiety, and increasing mental health problems have additionally been identified [25]. However, there is limited understanding of maternal and perinatal health following this disaster. There has been mixed evidence for immediate post-disaster changes in birth outcomes; some previous studies have found no increased proportions of low birthweight or preterm births in areas affected by the earthquake and tsunami [29], or in areas additionally affected by the nuclear disaster [30,31,32], in the first year post-disaster. However, there have also been findings of a slight increase in low birthweight neonates to women that had been 28–36 weeks pregnant at the time of the earthquake, in earthquake- and tsunami-affected areas [29], and increased proportions of low birthweight and preterm birth to women who conceived within six months post-disaster in areas affected by the Fukushima nuclear disaster [33]. However, despite the continuing social, psychological and physical health impacts of the nuclear disaster [25], there have been very few assessments to date of the long-term trends in birth outcomes in affected areas [32]; an area that calls for further elucidation.

**Figure 1:**
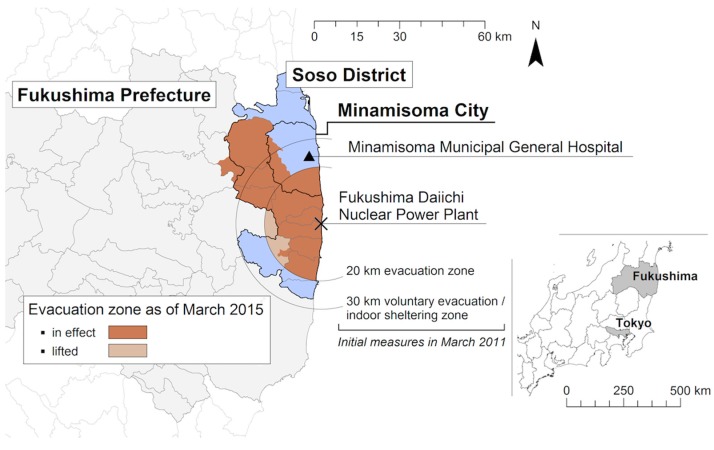
Map of Minamisoma Municipal General Hospital (MMGH) in relation to Fukushima Daiichi Nuclear Power Plant and evacuation zones.

In this institution-based study we evaluated data from Minamisoma Municipal General Hospital (MMGH), located 23 km from the plant (Figure 1), to investigate long-term trends in maternal and neonatal characteristics following the 2011 nuclear disaster. The objective of the present study is two-fold: to assess if there were long-term changes in birth outcomes following the Fukushima nuclear disaster, in comparison with pre-disaster baseline data, and to evaluate whether residential address at the time of the disaster, as a proxy measurement of evacuation, and avoidance of Fukushima food products, as a proxy measurement of radiation-related anxiety, were associated with any post-disaster birth outcomes.

## 2. Materials and Methods

### 2.1. Setting and Participants

All live singleton births at MMGH from April 2008 to 2015 were included in this study. On 12 March 2011, the 20 km radius of the power plant was classified as a restricted zone under mandatory evacuation orders by the central government of Japan [34]. On 15 March, those in the 20–30 km radius were ordered to shelter indoors, and on 25 March, this zone was classified as a voluntary evacuation area [25]. The mandatory evacuation zone has been under frequent updates, as described in previous reports [26], expanding to the northwest mountainous areas heavily affected by radioactive fallout. MMGH falls just outside of the mandatory evacuation zone, and serves areas significantly affected by the nuclear disaster. Although the Obstetrics and Gynecology Department of the hospital closed immediately after the disaster, it re-opened in April 2012. The time-period of this study therefore captures three years of post-disaster data (2012–2015) on births in this hospital, compared to the same length of period pre-disaster (2008–2011), defined in the format of Japanese fiscal years which begin in April and end in March of the following year.

### 2.2. Data Collection

Data on maternal characteristics and birth outcomes were extracted from the hospital’s patient records. Maternal characteristics included age at time of the birth, number of previous deliveries (parity) and residential address. Birth data of birthweight, gestational age at birth, mode of delivery (vaginal delivery or caesarean section), date of delivery, and sex of the neonate were collected.

### 2.3. Main Outcome Measures

The following two outcome measures were considered as primary birth outcomes of interest in this study: low birthweight (<2500 g at birth), and preterm birth (<37 weeks of gestation at birth).

### 2.4. Residential Area at the Time of the Disaster

The difficulty of defining maternal exposure to a disaster has been previously noted [9]. The present study uses residential area at the time of the disaster [9] to estimate evacuation experience as an indicator of maternal disaster exposure. For all mothers who delivered in the post-disaster period, data on residential address at the time of the disaster was extracted from hospital records. Mothers were then classified into four groups based on evacuation orders: (1) inside the mandatory evacuation zone; (2) inside the indoor sheltering/voluntary evacuation zone; (3) inside areas of Soso District under no evacuation orders; and (4) outside Soso District. For participants in the pre-disaster period, residential address at the time of delivery was classified in the same manner. The geographical scope of the evacuation orders during the study period is displayed in Figure 1. Soso District is specified in these classifications as it was significantly affected by the disasters, with areas falling in the mandatory, voluntary, and non-evacuation zones, and significant evacuation even in non-ordered areas; it is reported by the Japanese Ministry of Education, Culture, Sports, Science and Technology (MEXT) that at the time point of 15 March 2011, 102,882 Fukushima Prefecture residents had evacuated within or outside the Prefecture, and voluntary evacuees were estimated to account for 39.1% (40,256 people), leaving both voluntary evacuation zones and non-ordered areas [35].

### 2.5. Post-Disaster Food Purchasing Patterns

This study additionally used data on maternal food purchasing patterns. Since March 2012, all pregnant women under the care of MMGH have been encouraged to undergo free Whole Body Counter (WBC) internal radiation contamination screenings at MMGH during their pregnancies. An exposure risk assessment questionnaire is given at the time of WBC screenings, which contains items on methods of acquiring the following six food products: rice, meat, fish, produce, mushrooms and milk. Each item has four choices: (a) purchasing food products at a supermarket based on origin (Fukushima vs. non-Fukushima); (b) purchasing food products at a supermarket without consideration of origin; (c) using local farms or consuming home grown foods with radiation inspection; or (d) without it.

We extracted data on the food purchasing preferences of participants in the post-disaster period, with the hypothesis that avoidance of Fukushima products could be an indicator of radiation-related anxiety. If participants had undergone multiple WBC screenings during their pregnancy, questionnaire data were extracted from the screening closest to the date of delivery. In all WBC screenings of pregnant women from 2012 to 2015, there were no cases of detectable internal radiation contamination (detection limits with a 2-min scan: 210 Bq/body for Caesium-134 and 250 Bq/body for Caesium-137); therefore, internal radiation levels could not be considered as a variable for analysis in this study.

### 2.6. Statistical Analyses

We conducted two analyses. First, to evaluate any difference in the rates of post- versus pre-disaster birth outcomes, proportions of low birthweight and preterm birth were calculated for each period and compared, expressed as risk ratios (RRs).

Second, to examine any associations of these two outcomes (low birthweight and preterm birth) with residential area at the time of the disaster or food purchasing patterns, adjusted for potential covariates, we performed multivariate logistic regression analyses with the post-disaster data. For model building, variables initially entered into the regression models were chosen based on univariate analyses. Additional model selection was performed using backward-stepwise method with p-to-remove of >0.05. Backward-stepwise regression starts with all the candidate variables in the model and removes the least significant variables until all the remaining variables are statistically significant. Basic variables, such as year, maternal age at time of birth, sex of neonate, and the number of previous deliveries, as well as those of main interest in this study (i.e., residential address at the time of the disaster and post-disaster food patterns) were incorporated into the final model regardless of their statistical significance as long as stable models were obtained. The partial F-test was used to verify the entry and removal of variables from the model. Since some participants had more than one delivery at MMGH during the study period, the regression model included a random effect at individual level to control for the fact that the same individual’s data were correlated.

### 2.7. Ethics Approval

Ethics approval for this study was granted by the MMGH Institutional Review Board, reference number 27-21. Participant consent was not found to be necessary, as this was a retrospective analysis of hospital records. All data were anonymised prior to analysis.

## 3. Results

### 3.1. Characteristics of Study Participants

There were 1134 births recorded at MMGH from April 2008 through March 2015. Eleven sets of twins, three intentional abortions, four stillbirths and four miscarriages recorded as births were excluded, resulting in 1101 live singleton births (delivered from 1009 mothers) included in this study. By year, there were 236 births in 2008, 221 in 2009, 214 in 2010, 0 in 2011 (due to study institution closure, as mentioned above), 90 in 2012, 162 in 2013, and 178 in 2014.

**Figure 2:**
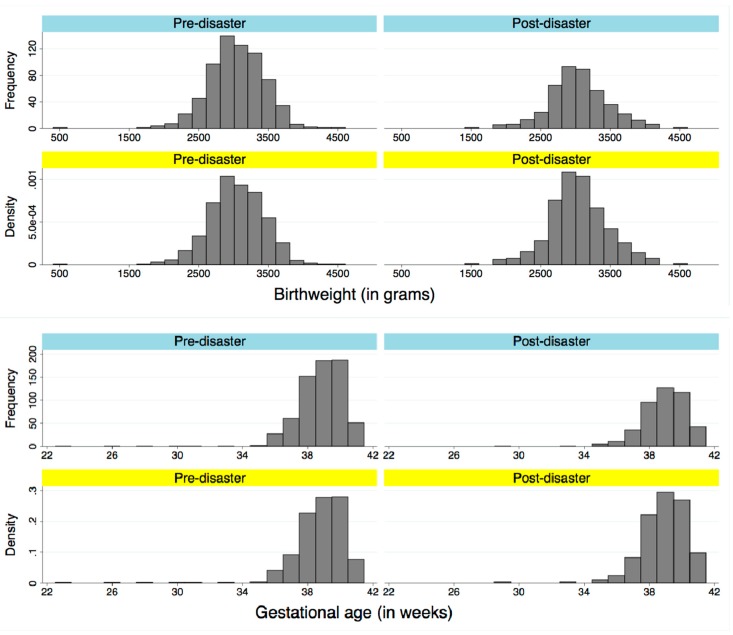
Pre- and post-disaster distribution of birthweight in grams and gestational age at delivery in weeks.

Maternal and neonatal characteristics by year are described in Table 1. There were no significant differences between years in the proportions of low birthweight or preterm births. The distributions of birthweight and gestational age at birth in pre- and post-disaster periods are displayed in Figure 2. The number of previous deliveries per mother significantly differed between years, with a pre-disaster decrease in mothers with two or more previous deliveries (and increase in mothers with 0 or 1 previous deliveries) in 2009, and a post-disaster increase in first-time mothers peaking in 2014 (*p* < 0.001). There were significant changes in maternal residential address patterns throughout years (*p* < 0.001) with post-disaster decreases in deliveries at MMGH by those who had been living outside Soso District or within the mandatory evacuation zone at the time of the disaster, alongside increases in those who had been living in areas under voluntary evacuation orders or areas of Soso District under no evacuation orders. Because there were few mothers aged <19 years old (zero in 2008, four in 2009, three in 2010, zero in 2011, one in 2012, six in 2013 and four in 2014), we were unable to categorize this potentially high-risk group; maternal age at birth was instead categorized as <35 and >35 years, and a significant increase in mothers >35 years of age was observed after the disaster (*p* < 0.05). Other variables, such as sex of neonate, mode of delivery, and season of delivery were not significantly different by year.

Of the 430 mothers included in the post-disaster period, 401 (93.3%) participated in the WBC screenings. Of the 29 study participants that did not undergo WBC screening, six had been living outside Soso District at the time of the disaster, and 19 delivered in the last year of the study period (2014–2015). Trends in food purchasing choices are outlined in the additional material, and indicate that avoidance of locally produced rice and produce significantly increased as years passed after the disaster (*p* < 0.05 and *p* < 0.01, respectively) (Table S1).

### 3.2. Risk Ratios of Low Birthweight Birth and Preterm Birth

Table 2 shows the post- (2012, 2013, and 2014) versus pre-disaster (baseline: 2008–2010) RRs of low birthweight and preterm birth, adjusted for maternal age and neonatal sex. There were no statistically significant increases or decreases in the rates of preterm birth or low birthweight in any year after the disaster. We conducted a sensitivity analysis by merging all 2012–2014 data into a single post-disaster format, and non-significant results were observed (merged post-disaster RR of low birthweight birth: 0.98, 95% Confidence interval (CI): 0.62–1.51, *p* = 0.93; and RR of preterm birth: 0.68, 95% CI: 0.38–1.21, *p* = 0.19).

### 3.3. Regression Analysis

Table 3 shows results of the regression analysis for post-disaster low birthweight. The final model considered year, sex of neonate, mode of delivery, maternal age, number of prior deliveries, and residential address at the time of the disaster. There were no statistically significant associations found with post-disaster low birthweight. This final model for low birthweight was not able to include the variable of post-disaster food purchasing patterns (which were not statistically significant) because of model instability. Sensitivity analyses were performed that constructed three different regression models in which data of 2008, 2009, and 2010 were considered as reference years; similar results were obtained (data not available). Similar results were obtained in the regression analysis for preterm birth (Table S2), in which the final model showed no statistical significance in the relationship between any variables and preterm birth.

The results of univariate analyses, showing no statistically significant associations between food purchasing patterns and low birthweight or preterm births, are displayed in Table S3.

## 4. Discussion

This study retrospectively assessed all live singleton births from 2008 to 2014 in a hospital serving areas affected by the 2011 Fukushima Daiichi Nuclear Power Plant Accident, finding no significant long-term changes in the prevalence of low birthweight or preterm births after the disaster, compared to a pre-disaster period. There were additionally no associations between residential address at the time of the disaster or food purchasing patterns, and post-disaster birth outcomes. We did confirm a substantial decrease in the number of births occurring at MMGH after the disaster, with no births in 2011 due to departmental closure, followed by gradually increasing numbers in each post-disaster year.

Previous studies on birth outcomes following the Fukushima disaster have produced mixed results, with some studies finding no significant changes in birth outcomes in areas affected by the nuclear disaster [30,31,32], and others finding increased proportions of low birthweight and preterm births [33]; however, most studies to date have only assessed outcomes within the first year of the disaster. The overall inconsistency within results from Fukushima, and between results from Fukushima and other disasters where increases in low birthweight or preterm births have been predominant indicate that the effects of this disaster may differ from those observed in other settings [9,10,11,12,13]. In order to interpret inconsistencies between results of post-disaster studies on birth outcomes, it has been noted that clear assessment of the pathways between disasters and outcomes is crucial [9,21]. Two commonly proposed pathways to post-disaster changes in population birth outcomes are environmental exposures and psychological stress [9,21], and we took particular methodological considerations of these factors in our study, as outlined below.

In terms of environmental exposures, nuclear disasters are rare and understudied events that present the danger of exposing populations to radioactive materials. However, impacts of nuclear disasters on population birth outcomes are not well studied, and likely to vary by the scale of each disaster. Studies after the Chernobyl nuclear disaster in 1986 indicate mixed evidence for a small increase in congenital anomalies, yet overall little effect on most pregnancies [9,36,37]. Radiation related health risks in Fukushima have been found to be significantly less than those in Chernobyl due to lower exposure doses [38], and the United Nations Scientific Committee on the Effects of Atomic Radiation (UNSCEAR) has predicted no deterministic effects of radiation exposure to the general public in Fukushima [38]. In the present study, none of the post-disaster mothers who underwent WBC screening (*n* = 401, 93.2%) had detectable levels of internal radiation contamination, and we therefore find it unlikely that radiation exposure would have had any effect on birth outcomes in this study. However, the Fukushima disaster has led to social disruption and public concern for radiation exposure [25] which may pose its own risks to maternal and perinatal health; a previous study after the Chernobyl accident found radiation-related anxiety, not radiation itself, to be associated with earlier births [39].

Psychological stress is a frequently reported pathway from disaster occurrence to changes in population birth outcomes [9,21,40]. We attempted to capture potential effects of stress by categorizing participants based on residential address at the time of the disasters and food purchasing patterns as indicators of evacuation and anxiety about radiation, respectively. Further, following findings on the effects of pre-conception stressful life events on birthweight [23,24] and the long-term increases in adverse birth outcomes seen after the Red River Catastrophic Flood [10] and the 11 September attacks [22], we included three years in the post-disaster period to assess for any long-term changes. Our finding that these indicators were not significantly associated with birth outcomes may suggest that disaster experience in itself does not qualify as a stressful life event with long-term effects on reproductive health. However, it is possible that our approach of measuring food purchasing patterns and residential address at the time of the disaster were unable to accurately capture pathways from disaster exposure to birth outcomes. Food purchasing patterns may be limited as a proxy measurement for anxiety, as they are generally linked with household socioeconomic factors and local food availability, two factors that may be affected by the disaster, yet were not possible to assess in this study. It should also be noted that while 93.2% of the post-disaster participants in this study underwent WBC screening and completed the food questionnaire, out of the 29 that did not complete it, six had been living outside Soso District at the time of the disaster, and 19 had delivered in the last year of the study (2014), meaning that food questionnaire results may be most representative of the population living within Soso District in 2012 and 2013. In terms of evacuation, we should acknowledge that movement after the disaster may have also been influenced by socioeconomic factors, meaning that there may have been socioeconomic differences between those who were able to evacuate and those who remained. Future evaluation of whether birth outcomes were patterned by socioeconomic factors, in both Fukushima and other disaster settings, would be of great benefit to begin filling this evidence gap that was out of the scope of our study.

This is not the first time that contradictory results on birth outcomes after disasters have been observed. In addition to some inconsistency in findings between different disasters [9,21], there have also been contradictory findings after the same disaster, as seen in the literature on Hurricane Katrina [11,15,17,41]. Recent studies on Hurricane Katrina have suggested that rapid population changes and differing risk profiles of the remaining population may have contributed to null findings, or even apparent reductions in risk of adverse birth outcomes [15,41], highlighting the need for disaster effects on birth outcomes to be considered in relation to potential population changes [15]. In this regard, we must recognize that large population shifts in Fukushima Prefecture have been documented since the 2011 disaster [42], and Minamisoma City in particular has experienced dramatic population loss, from 71,561 to approximately 10,000 within the first month of the disaster [43]. There has been slow population return to the city after the disaster, particularly in adult women [44]. We can speculate that one reason for null results observed in this study may have been the risk profile of the post-disaster population, which may have differed from the pre-disaster population (i.e., those at the highest risk of adverse birth outcomes may have been unable to return to the city after evacuation, as was speculated after Hurricane Katrina [15], and thus would not have been included in this study). The potential for population changes to influence results of birth outcome studies following disasters further underscores the need to understand any socioeconomic shifts in pre- and post-disaster populations, and how post-disaster adverse birth outcomes may relate to underlying population risks, in future studies.

Although this study found no changes in birth outcomes after the Fukushima nuclear disaster, our results suggest post-disaster changes in maternal demographics. There were statistically significant increases in the proportions of first-time mothers (*p* < 0.001), and in the proportions of mothers >35 years of age after the disaster (*p* < 0.05) (Table 1). This change is likely to be related to post-disaster population shifts as discussed above [44], with many women of reproductive age leaving to live elsewhere (either temporarily or permanently), and may also reflect a decision to delay childbirth on the part of women who remained in the area, as suggested by increased maternal age in the post-disaster period. Economic instability, community tensions and separation of families are issues that have been observed in post-disaster Fukushima [25,45], and all could have reduced social and economic resources available to women in disaster-affected areas—changes which could be speculated to have impacted fertility decisions being made in the study context. While it is unclear why the proportions of mothers with previous deliveries decreased (and first-time mothers increased) after the disaster, particularly in 2014 (Table 1), it could be hypothesized that anxiety or fear of radiation [46] may have influenced fertility decisions, potentially in different ways between women who already had children and those who did not. We also should consider that there may have been specific mental health impacts of the disaster to mothers; a recent study found high rates of depressive symptoms among mothers who were pregnant in 2010 or 2011 in Fukushima Prefecture [47], with particularly high rates in Soso District compared to other areas of Fukushima [47]. Depressive symptoms in women with deliveries around the time of the disaster may be related to the lower proportions of repeat pregnancies observed in the post-disaster period of the present study, as mothers experiencing depressive symptoms may have been less likely to want or try for repeat pregnancies. However, there is still limited information on the drivers of fertility decisions and maternal demographics following the Fukushima disaster, and nuclear disasters in general, and these areas deserve further research beyond our speculations here.

Japan is the most rapidly ageing country in the world, and had fertility rates below replacement levels since before the 2011 triple disaster [48]. We could not find any previous research that has discussed the ways in which disaster impacts on fertility patterns and birth outcomes may differ in baseline low fertility settings compared to high fertility settings, and we suggest that additional research in this area may be valuable, particularly as disasters are expected to happen more frequently in the future [49], and will more often hit low fertility countries as they continue to increase. The pathways of disasters effects on birth outcomes are still not conclusively understood, and could be hypothesized to function differently in low fertility vs. high fertility contexts. In this regard, Japan is representative of the global phenomenon of population ageing and declining fertility rates [48], and further assessment of the predictors of post-disaster fertility trends and birth outcomes in this context may be useful.

## 5. Strengths and Limitations

There are limitations to this study. First, we were unable to adjust analyses for maternal risk characteristics for low birthweight such as smoking and alcohol consumption, in addition to socioeconomic characteristics, because of limitations in data availability. Second, we did not have any data from April 2011 through March 2012, due to the closure of the Obstetrics and Gynecology Department in the study institution. Therefore, it was not possible to assess for any changes in birth outcomes in those who were pregnant at the time of the disaster (March 2011). Our lack of immediate post-disaster data may have contributed to the null results, and may not be directly comparable to studies that have assessed immediate post-disaster birth outcomes. The sample size was small for the analyses conducted, and although the data for this study comes from one institution, there are potential differences between our pre- and post-disaster samples that should be acknowledged as they could have caused sampling bias. As presented in the Discussion Section, post-disaster evacuation may have been patterned by socioeconomic factors, which could have influenced the population composition of those remaining in Soso District and thus participating in the post-disaster period of this study. It is further possible that disaster-related psychosocial stress may disproportionately affect women with low- or high-risk pregnancies, or those with lower or higher socioeconomic status, yet we were unable to assess for such characteristics due to limited data availability. For these reasons, we cannot rule out the possibility that sampling bias may have masked any real associations between the disaster and low birthweight or preterm birth.

However, alongside these limitations, this study has unique strengths. Methodological investigation of evacuation and food purchasing patterns, in addition to a prolonged study period, are points that could be informative to future research. Although efficacy of food purchasing preferences as a measurement tool was limited in this study, we suggest that open discussion of the methodological process undertaken here may be of use to future research in disaster settings; in essence, this is not only a public health study but also an account of the exploratory methods undertaken in a data-constrained post-disaster context. We suggest that there is a great need for in-depth exploration of the pathways to adverse birth outcomes, and the potential for socioeconomic patterning, in future studies.

## 6. Conclusions

The prevalence of low birthweight and preterm births did not significantly change in a hospital affected by the Fukushima Daiichi Nuclear Power Plant Accident, and there were no statistically significant associations between these birth outcomes and evacuation or food purchasing patterns in the post-disaster period. These results are inconsistent with previous findings on associations between disasters and adverse birth outcomes, and call for further research, particularly on the mechanisms by which disaster effects on maternal and perinatal health may be mediated.

## Figures and Tables

**Table 1:** Maternal and neonatal demographic characteristics by year (*n*, %).

Variables	2008	2009	2010	2012	2013	2014	*p*-Value for Percentage Difference *
Low Birthweight							0.67
No	217 (92.0)	203 (91.9)	202 (94.4)	85 (94.4)	152 (93.8)	161 (90.5)	
Yes	19 (8.1)	18 (8.1)	12 (5.6)	5 (5.6)	10 (6.2)	17 (9.6)	
Preterm Birth							0.51
No	223 (94.5)	212 (95.9)	201 (93.9)	88 (97.8)	153 (94.4)	173 (97.2)	
Yes	13 (5.5)	9 (4.1)	13 (6.1)	2 (2.2)	9 (5.6)	5 (2.8)	
Sex of Neonate							0.37
Male	120 (51.3)	104 (47.7)	119 (56.1)	40 (44.9)	77 (47.8)	94 (53.1)	
Female	114 (48.7)	114 (52.3)	93 (43.9)	49 (55.1)	84 (52.2)	83 (46.9)	
Mode of Delivery							0.26
Vaginal delivery	183 (77.5)	179 (81.0)	158 (73.8)	75 (83.3)	123 (75.9)	131 (73.6)	
Caesarean section	53 (22.5)	42 (19.0)	56 (26.2)	15 (16.7)	39 (24.1)	47 (26.4)	
Maternal Age (year)							<0.05
–35]	201 (85.2)	181 (81.9)	183 (85.5)	67 (74.4)	122 (75.3)	143 (79.2)	
(35–	35 (14.8)	40 (18.1)	31 (14.5)	23 (25.6)	40 (24.7)	37 (20.8)	
Number of Previous Deliveries							<0.001
0	44 (18.6)	94 (42.5)	90 (42.1)	34 (37.8)	74 (45.7)	97 (54.5)	
1	31 (13.1)	86 (38.9)	88 (41.1)	39 (43.3)	59 (36.4)	52 (29.2)	
2 or more	161 (68.2)	41 (18.6)	36 (16.8)	17 (18.9)	29 (17.9)	29 (16.3)	
Residential Area ^†^							<0.001
Inside the mandatory evacuation zone	84 (35.6)	92 (41.6)	79 (36.9)	11 (12.2)	16 (9.9)	26 (14.6)	
Inside the sheltering/voluntary evacuation zone	44 (18.6)	39 (17.7)	40 (18.7)	28 (31.1)	78 (48.2)	79 (44.4)	
Inside areas of Soso District under no evacuation orders	32 (13.6)	28 (12.7)	23 (10.8)	32 (35.6)	53 (32.7)	48 (27.0)	
Outside Soso District	76 (32.2)	62 (28.1)	72 (33.6)	19 (21.1)	15 (9.3)	25 (14.0)	
Season of Delivery							0.25
Spring	57 (24.2)	66 (29.9)	57 (26.6)	15 (16.7)	36 (22.2)	52 (29.2)	
Summer	59 (25.0)	55 (24.9)	58 (27.1)	18 (20.0)	39 (24.1)	43 (24.2)	
Autumn	60 (25.4)	45 (20.4)	52 (24.3)	26 (28.9)	40 (24.7)	42 (23.6)	
Winter	60 (25.4)	55 (24.9)	47 (22.0)	31 (34.4)	47 (29.0)	41 (23.0)	

* Chi-squared test (or Fisher’s exact test, when there were fewer than five observations). ^†^ For 2008–2010, this indicates place of residence at the time of delivery, and for 2012–2014, indicates place of residence at the time of the Fukushima nuclear disaster.

**Table 2:** Post- versus pre-disaster risk ratios of low birthweight and preterm birth, adjusted for maternal age and neonatal sex.

Birth Outcome	Risk Ratio	95% CI	*p*-Value
Low Birthweight			
2012	0.71	0.29–1.75	0.46
2013	0.80	0.42–1.55	0.52
2014	1.28	0.76–2.17	0.35
Preterm Birth			
2012	0.40	0.10–1.64	0.20
2013	1.01	0.49–2.05	0.99
2014	0.52	0.21–1.30	0.16

**Table 3:** Regression model for post-disaster low birthweight (95% CI).

Variable	Odds Ratio	95% CI	*p*-Value
Year			
2012	reference		
2013	0.83	0.15–4.41	0.82
2014	1.69	0.38–7.63	0.44
Sex of Neonate			
Male	reference		
Female	3.15	0.77–12.87	0.11
Mode of Delivery			
Vaginal delivery	reference		
Caesarean section	4.27	0.81–22.47	0.09
Maternal Age (year)			
–35]	reference		
(35–	1.06	0.28–4.01	0.93
Number of Previous Deliveries			
0	reference		
1	0.73	0.20–2.57	0.62
More than 2	0.56	0.10–3.08	0.51
Residential Area			
Inside the mandatory evacuation zone	0.91	0.11–7.17	0.93
Inside the sheltering/voluntary evacuation zone	1.00	0.19–5.31	1.00
Inside areas of Soso District under no evacuation orders	0.54	0.08–3.60	0.52
Outside Soso District	reference		

## Supplementary Materials

The following are available online at www.mdpi.com/1660-4601/14/5/542/s1, Table S1: Post-disaster food purchasing patterns; Table S2: Regression model for post-disaster preterm birth; Table S3: Number (percentage) of low birthweight and preterm births by food purchasing patterns.

## Availability of Data and Material

The datasets generated and analysed during the current study are not publicly available as they contain information that could compromise research participant privacy.
