# Supplementary material for: Birth Outcomes after the Fukushima Daiichi Nuclear Power Plant Disaster: A Long-Term Retrospective Study
Source: Int J Environ Res Public Health. 2017 May 19;14(5):542. doi: 10.3390/ijerph14050542 (PMC5451992; doi:10.3390/ijerph14050542)
Supplement: Supplementary file 1 [file ijerph-14-00542-s001.zip › ijerph-186009-supplementary/IJERPH Supplementary files/IJERPH Supplementary table 1.pdf]

Supplementary Table 1: Post-disaster food purchasing patterns (n, %)

	Year	Category*				P-value†
		(a)	(b)	(c)	(d)	
Rice	2012	46 (59.7)	8 (10.4)	18 (23.4)	5 (6.5)	<0.05
	2013	79 (56.4)	27 (19.3)	33 (23.6)	1 (0.7)	
	2014	99 (60.7)	27 (16.6)	37 (22.7)	0 (0.0)	
Meat	2012	61 (70.1)	26 (29.9)	0 (0.0)	0 (0.0)	0.45
	2013	87 (62.1)	53 (37.9)	0 (0.0)	0 (0.0)	
	2014	104 (63.8)	59 (36.2)	0 (0.0)	0 (0.0)	
Fish	2012	65 (74.7)	22 (25.3)	0 (0.0)	0 (0.0)	0.77
	2013	100 (71.9)	39 (28.1)	0 (0.0)	0 (0.0)	
	2014	120 (73.6)	41 (25.2)	2 (1.2)	0 (0.0)	
Produce	2012	43 (57.3)	18 (24.0)	7 (9.3)	7 (9.3)	<0.01
	2013	91 (66.4)	36 (26.3)	7 (5.1)	3 (2.2)	
	2014	105 (71.9)	35 (24.0)	6 (4.1)	0 (0.0)	
Mushrooms	2012	60 (69.0)	27 (31.0)	0 (0.0)	0 (0.0)	0.82
	2013	96 (68.6)	44 (31.4)	0 (0.0)	0 (0.0)	
	2014	117 (71.8)	46 (28.2)	0 (0.0)	0 (0.0)	
Milk	2012	55 (64.7)	28 (32.9)	1 (1.2)	1 (1.2)	0.22
	2013	74 (52.9)	64 (45.7)	2 (1.4)	0 (0.0)	
	2014	98 (60.1)	64 (39.3)	1 (0.6)	0 (0.0)	

* (a) Selecting food products at a supermarket based on origin (Fukushima vs. non-Fukushima)
(b) Selecting food products at a supermarket without considering origin
(c) Using local farms or homegrown foods with radiation inspection
(d) Using local farms or homegrown foods without radiation inspection
†Fisher's exact test for comparing percentages
