# Supplementary material for: Birth Outcomes after the Fukushima Daiichi Nuclear Power Plant Disaster: A Long-Term Retrospective Study
Source: Int J Environ Res Public Health. 2017 May 19;14(5):542. doi: 10.3390/ijerph14050542 (PMC5451992; doi:10.3390/ijerph14050542)
Supplement: Supplementary file 1 [file ijerph-14-00542-s001.zip › ijerph-186009-supplementary/IJERPH Supplementary files/IJERPH Supplementary table 2.pdf]

Supplementary Table 2: Regression model for post-disaster preterm birth (95% CI)

Variable	Odds ratio	95% CI	P-value
Year			
2012	Ref.		
2013	2.20	0.45–10.77	0.33
2014	1.04	0.19–5.70	0.96
Sex of neonate			
Male	Ref.		
Female	0.56	0.19–1.59	0.27
Maternal age [year]			
–35]	Ref.		
(35–	1.02	0.30–3.39	0.98
Number of previous deliveries			
0	Ref.		
1	0.41	0.11–1.56	0.19
More than 2	1.08	0.31–3.81	0.91
Residential area			
Inside the mandatory evacuation zone	1.24	0.07–20.93	0.88
Inside the sheltering/voluntary evacuation zone	2.38	0.28–20.37	0.43
Inside areas of Soso District under no evacuation orders	2.61	0.29–23.16	0.39
Outside Soso District	Ref.		
