# Supplementary material for: Birth Outcomes after the Fukushima Daiichi Nuclear Power Plant Disaster: A Long-Term Retrospective Study
Source: Int J Environ Res Public Health. 2017 May 19;14(5):542. doi: 10.3390/ijerph14050542 (PMC5451992; doi:10.3390/ijerph14050542)
Supplement: Supplementary file 1 [file ijerph-14-00542-s001.zip › ijerph-186009-supplementary/IJERPH Supplementary files/IJERPH Supplementary table 3.pdf]

Supplementary Table 3: Number (percentage) of low birthweight and preterm births by food purchasing patterns*

	Rice		Meat		Fish		Produce		Mushrooms		Milk	
Birthweight	(2500-	-2500]	(2500-	-2500]	(2500-	-2500]	(2500-	-2500]	(2500-	-2500]	(2500-	-2500]
(1)	205 (91.5)	19 (8.5)	236 (93.7)	16 (6.4)	267 (93.7)	18 (6.3)	223 (93.3)	16 (6.7)	254 (93.0)	19 (7.0)	211 (93.0)	16 (7.1)
(2)	58 (93.6)	4 (6.5)	125 (90.6)	13 (9.4)	91 (89.2)	11 (10.8)	81 (91.0)	8 (9.0)	107 (91.5)	10 (8.6)	144 (92.3)	12 (7.7)
(3)	83 (94.3)	5 (5.7)	0 (0.0)	0 (0.0)	2 (100.0)	0 (0.0)	18 (90.0)	2 (10.0)	0 (0.0)	0 (0.0)	4 (100.0)	0 (0.0)
(4)	5 (83.3)	1 (16.7)	0 (0.0)	0 (0.0)	0 (0.0)	0 (0.0)	10 (100.0)	0 (0.0)	0 (0.0)	0 (0.0)	0 (0.0)	1 (100.0)
P-value†	0.56		0.31		0.30		0.72		0.67		0.14	
Gestational term	Full term	Preterm	Full term	Preterm	Full term	Preterm	Full term	Preterm	Full term	Preterm	Full term	Preterm
(1)	217 (96.9)	7 (3.1)	245 (97.2)	7 (2.8)	278 (97.5)	7 (2.5)	231 (96.7)	8 (3.4)	266 (97.4)	7 (2.6)	220 (96.9)	7 (3.1)
(2)	61 (98.4)	1 (1.6)	133 (96.4)	5 (3.6)	97 (95.1)	5 (4.9)	86 (96.6)	3 (3.4)	112 (95.7)	5 (4.3)	151 (96.8)	5 (3.2)
(3)	84 (95.5)	4 (4.6)	0 (0.0)	0 (0.0)	2 (100.0)	0 (0.0)	20 (100.0)	0 (0.0)	0 (0.0)	0 (0.0)	4 (100.0)	0 (0.0)
(4)	6 (100.0)	0 (0.0)	0 (0.0)	0 (0.0)	0 (0.0)	0 (0.0)	10 (100.0)	0 (0.0)	0 (0.0)	0 (0.0)	1 (100.0)	0 (0.0)
P-value†	0.72		0.76		0.36		1.00		0.36		1.00	

* (1) Selecting food products at a supermarket based on origin (Fukushima vs. non-Fukushima)
(2) Selecting food products at a supermarket without considering origin
(3) Using local farms or homegrown foods with radiation inspection
(4) Using local farms or homegrown foods without radiation inspection

† Fisher's exact test for comparing percentages
